# Laparoscopic surgery for splenic injuries in the era of non-operative management: current status and future perspectives

**DOI:** 10.1007/s00595-020-02177-2

**Published:** 2020-11-16

**Authors:** Luigi Romeo, Francesco Bagolini, Silvia Ferro, Matteo Chiozza, Serafino Marino, Giuseppe Resta, Gabriele Anania

**Affiliations:** 1 Department of Morphology, Surgery and Experimental Medicine, Sant’Anna University Hospital, University of Ferrara, Via Aldo Moro 8, 44124 Ferrara, Italy; 2 Department of Surgery, Surgery 1 Unit, Sant’Anna University Hospital, Via Aldo Moro 8, 44124 Ferrara, Italy

**Keywords:** Splenic injury, Splenic trauma, Abdominal trauma, Laparoscopic splenectomy

## Abstract

The spleen is one of the organs most commonly injured by blunt abdominal trauma. It plays an important role in immune response to infections, especially those sustained by encapsulated bacteria. Nonoperative management (NOM), comprising clinical and radiological observation with or without angioembolization, is the treatment of choice for traumatic splenic injury in patients who are hemodynamically stable. However, this strategy carries a risk of failure, especially for high-grade injuries. No clear predictors of failure have been identified, but minimally invasive surgery for splenic injury is gaining popularity. Laparoscopic surgery has been proposed as an alternative to open surgery for hemodynamically stable patients who require surgery, such as after failed NOM. We reviewed research articles on laparoscopic surgery for hemodynamically stable patients with splenic trauma to explore the current knowledge about this topic. After presenting an overview of the treatments for splenic trauma and the immunological function of the spleen, we try to identify the future indications for laparoscopic surgery in the era of NOM.

## Introduction

The spleen and liver are the organs most frequently injured in penetrating and blunt abdominal trauma [1]. The treatment of splenic trauma changed greatly during the second half of the last century, switching from surgical treatment to non-operative management (NOM). The main driver of NOM implementation was acknowledgment of the important role played by the spleen in the immunologic response, together with the avoidance of surgery and its related risks. The loss of immunologic function of the spleen after splenectomy increased susceptibility to infections, especially those caused by encapsulated bacteria, which can lead to severe and rapidly progressive infection, named “overwhelming post-splenectomy infection” (OPSI). This complication is associated with mortality rates as high as 50% [2]. The most common causative pathogens are *Strepstococcus pneumoniae*, *Nesserya meningitidis,* and *Hemophilus influenza.* Thus, vaccines for these bacteria are recommended for patients who undergo splenectomy, together with patient education and prompt antibiotic administration for any suspected infection. However, concerns remain about NOM for high-grade splenic injury, because of its high failure rate [3].

Laparoscopy has revolutionized surgery since its introduction in the late 1980s, reducing surgical trauma, recovery time, and hospitalization. Currently, laparoscopic surgery is applied widely in many surgical fields. Its introduction in emergency surgery started late, because patients who need emergency surgery are often hemodynamically unstable. However, the continued evolution of materials and technologies, together with the improved laparoscopic skills of surgeons, has led to laparoscopic surgery being used more frequently in emergency situations. In particular, the use of laparoscopy both with diagnostic and therapeutic intent in hemodynamically stable trauma patient has increased in recent years [4]. In the early 1990s, some authors reported performing laparoscopic surgery for splenic trauma. Subsequent papers on this subject were published, ranging from single case reports to retrospective studies comparing open and laparoscopic procedures for splenic injuries.

We reviewed the literature on laparoscopic surgery for splenic traumatic injuries to provide a focused and updated summary, and to try to identify prospectively the indications for laparoscopic surgery for splenic trauma that could develop in the era of NOM.

## Current treatment of splenic trauma

Until the immunological function of the spleen was understood, injured spleens were treated with splenectomy, based on the idea that the natural evolution of splenic injuries was splenic rupture. However, in the 1960s, better knowledge about the functions of the spleen in immune response and the recognition and definition of Overwhelming Post-Splenectomy Infection (OPSI) [5–7], induced surgeons to consider preserving injured spleens. This idea led to a change in the therapeutic paradigm from surgical treatment to conservative management. Advances in radiology technologies, both diagnostic (such as computed tomography (CT) and ultrasound) and therapeutic (such as interventional radiology), also played a pivotal role in this revolution in the treatment of splenic trauma. In fact, radiologic progress has allowed physicians to assess splenic involvement in trauma more accurately and even to treat bleeding while avoiding splenectomy in selected cases [8]. In addition to technological progress, the scientific community has provided a better definition of the severity of injuries after trauma through the American Association for Surgery of Trauma Organ Injury Scaling (AAST-OIS) [9]. This classification enables all physicians involved in trauma care to standardize studies about this topic and indications according to the injury grade. This scale was updated in 2018 [10].

As result of the progress and changes, NOM is now considered the gold standard for hemodynamically stable patients with splenic injuries. NOM consists of clinical and radiological observation, with or without the aid of angioembolization. According to the most recent guidelines of WSES [11], NOM should be chosen for all hemodynamically stable splenic injuries that do not require laparotomy for other reasons, reserving emergency splenectomy for hemodynamically unstable patients, regardless of splenic grade of injury. However, NOM for high-grade (AAST grade IV−V) splenic injuries is still under debate, because of the high failure rate reported [12]. A systematic review by Cirocchi [3], concluded that NOM is widely accepted as the standard of treatment for grade I and II splenic injuries, but there is no consensus about its safety for higher-grade injuries. Angioembolization is an effective adjunct to NOM in case of contrast blush on CT scan or splenic pseudoanesurysms. Some studies have demonstrated that it can improve the results of NOM [12, 13]. Several authors have advocated the use of prophylactic angioembolization for high-grade splenic injury [14–16], but this topic remains under debate [11].

Many factors have been related to failure of NOM. Splenic injury grade and the severity of overall injuries are considered the most important predictive factor for failure of NOM. In fact, many studies show that the more severe the splenic injury, the higher the risk of failure [17–19]. Some studies [20–23] suggest that age is a predictor of failure after NOM, but others deny this association [24, 25]. Other factors associated with failure are the amount of hemoperitoneum [17, 26] and the need for transfusion [19, 23].

## Laparoscopic splenectomy for trauma

In 2001, Ren et al. [27] reported a splenic traumatic injury managed with hand-assisted laparoscopy, but did not describe either the hemodynamic status of the patient or the outcomes of the procedure. Despite the limited information provided, this is the first reported case of post-traumatic splenectomy using a laparoscopic technique. About 2 years later, Basso et al. [28] reported a case of totally laparoscopic splenectomy performed in a 31-year-old man, 10 days after the trauma, following failure of NOM due to ongoing bleeding. All the procedures were performed with the patient supine because of an acetabular fracture that did not allow for lateral positioning. They did not report any postoperative complications.

Many cases of laparoscopic splenectomy have been reported since, with different indications (Table 1). In 2006, the case of a young man with grade V splenic injury treated with laparoscopic splenectomy was reported [29]. This patient also presented with a synchronous kidney injury, which, according to the authors, could have complicated NOM. Given the hemodynamic stability of the patient and after discussing the different treatment options with him, he underwent laparoscopic splenectomy, followed by an uneventful postoperative course. Agarwal [30] reported a laparoscopic splenectomy in a 46-year-old man admitted with a grade III splenic injury with mild perisplenic collection after a car accident. NOM was attempted initially, but his hemoglobin level dropped within 1 day and a repeat CT scan showed an increase in the perisplenic fluid collection. This prompted the author to consider splenectomy and the hemodynamic stability of the patient allowed the laparoscopic approach. Ayiomamitis [31] and Rolton [32] reported two other cases of successful laparoscopic splenectomy. The first was a 76-year-old woman with a hemodynamically stable grade III splenic injury and associated left rib fractures, who suffered hypotension with a drop in hemoglobin 26 h after admission. This was responsive to fluid infusion, but the surgeon decided to perform laparoscopic splenectomy, considering also that the patient refused blood transfusion. The operative time was 65 min and the postoperative course was complicated by left basal pneumonia. She was discharged on postoperative day 16. The second case was of a 24-years-old woman admitted after a high-speed motor vehicle accident during her 27th week of gestation. Chest X-rays showed a left diaphragmatic rupture and she was transferred to the operating room for laparoscopic repair. Intraoperatively, the surgeon found a splenic capsular injury with active bleeding from the hilar vessels and performed a laparoscopic splenectomy. She had an uneventful recovery and the pregnancy was completed.

**Table 1:** Summary of case reports of laparoscopic splenectomy after traumatic injury

Authors	Years	indications	AAST–OIS grade	Hemodynamic status	Embolization	Associated injuries	Complications	Mortality
Basso et al.	2003	Delayed splenic rupture 10 days after trauma	4	Stable	No	Fractures of right humerus, left acetabulum,	No	No
Dikkani et al.	2006	Splenic laceration after falling trauma	5	Stable	No	Grade 2 renal injury	No	No
Pucci et al.	2007	Failure of NOM with selective embolization for splenic post-traumatic pseudoaneurysm	Not reported	Stable	Yes	None	No	No
Ayiomamitis et al.	2008	Splenic injury in a Jehovah’s Witness patient with Hb drop after initial NOM	3	Stable	No	Fractures of the left seventh and eighth ribs	Left basal pneumonia	No
Ransom et al.	2008	Failure of NOM with central embolization (Hb decrease and ongoing tachycardia)	5	Stable	Yes	Not reported	No	No
Agarwal	2009	Enlarging splenic hematoma and decreasing Hb levels	3	Stable	No	Fractures of left eighth and ninth ribs	No	No
Rolton et al.	2009	Splenic injury discovered during laparoscopic repair of a diaphragmatic hernia in a pregnant	Not reported	Stable	No	left fifth rib, left radius and ulna, and left sided	No	No
Fan et al.	2011	Vital signs deterioration despite resuscitation in splenic injury after fall from a 5-m height	3	Not reported	No	None	No	No

*AAST–OIS *American Association for the Surgery of Trauma–organ injury scale

Two series of patients treated with splenectomy after blunt injury were published during the same period. The first describes four hemodynamically stable patients treated with laparoscopic splenectomy after a drop in the hematocrit level during NOM, representing failure of the NOM [33]. The second reports 11 cases of laparoscopic surgery after splenic blunt trauma in patients with hemodynamic stability [34]. Six of these 11 patients underwent splenectomy. No complications were reported, and only one needed intraoperative conversion to subcostal minilaparotomy, to control bleeding. However, the indications for surgery were not specified, and the need for surgery in these patients could be disputed. Both series state that laparoscopic splenectomy is a viable alternative to open surgery, if performed by surgeons experienced in laparoscopic procedures.

Other series were published in 2010 [35] and 2013 [36]. Carobbi et al. [35] reported a series of 12 patients who underwent laparoscopic surgery for splenic traumatic injury. Ten of these patients underwent splenectomy. The parameters considered when selecting the treatment were the Injury Severity Score, the degree of splenic injury, the Glasgow Coma Scale, and the amount of hemoperitoneum. Patients with an Injury severity score of 20 or higher, a high-grade splenic injury, and hemoperitoneum extending to at least two recesses were considered for surgery. If the patient had a Glasgow Coma Scale higher than 10, they were considered suitable for laparoscopy. The reported outcomes were encouraging: there was no postoperative mortality or morbidity, none of the patients required conversion to laparotomy, and the median surgical time and postoperative length of stay were 120 min and 4 days, respectively. Yahya and colleagues [36] reported their experience at a hospital in Libya. None of the patients had CT scans done on admission, but the decision to perform laparoscopic exploration was based on clinical and sonographic findings. They reported a heterogeneous series of 18 patients affected by splenic injuries and treated very differently. Three of these patients underwent laparoscopic splenectomy and one required conversion to laparotomy after laparoscopic exploration because of complete splenic avulsion. A single case of transumbilical single-incision splenectomy for traumatic splenic injury in a 19-year-old man after a fall was also reported [37]. No conversion to open surgery was required and there were no postoperative complications.

Some authors have reported successful laparoscopic splenectomy after failure of NOM with endovascular splenic artery embolization for ongoing bleeding [38, 39]. Ransom and Kavic [40] reported a retrospective series of 11 patients who underwent splenectomy for ongoing bleeding or splenic abscess after embolization. Four of these patients underwent laparoscopic splenectomy, while seven were treated with laparotomy. All patients were hemodynamically stable. Regardless of the indication for splenectomy, the laparoscopic group had a longer operative time, but a shorter hospital stay.

### Laparoscopic versus open splenectomy

Studies published in recent years compare open and laparoscopic surgery for traumatic splenic injury. Ermolov et al. [41] reported a comparative analysis of 42 patients with hemodynamically stable grade III blunt splenic injury, 23 of whom underwent laparoscopic splenectomy, whereas 19 underwent open splenectomy. The indications for laparoscopic splenectomy were a spleen laceration greater than 3 cm with moderate ongoing bleeding and an expanding hematoma. The patient demographics and severity of injury were similar in the two groups. The laparoscopic group had a longer operative time, but shorter paralytic ileus and bedrest time.

Shamin reported the results of an analysis of the National Trauma Database, comparing 113 patients who underwent laparoscopic splenectomy with 25,408 patients who underwent open surgery for both blunt and penetrating trauma [42]. In this study, an ISS lower than 25 and a systolic blood pressure higher than 140 mm Hg were predictive factors for laparoscopic splenectomy. The two groups did not show significant differences in in-hospital mortality, in-hospital length of stay, and major complications. The study concluded that laparoscopic splenectomy is a feasible and safe if performed by experienced surgeons, with similar outcomes to the open procedure. Another retrospective comparative study published in 2017 enrolled 52 patients who underwent splenectomy for splenic trauma: 41 procedures were performed with open surgery, while 11 were performed laparoscopically [43]. The two groups did not differ in demographics or admission parameters, except for a lower GCS and Base Excess in the laparotomy group. The most common indication for laparoscopic splenectomy was failure of NOM and embolization. The laparoscopic group had less intraoperative blood loss and a longer operative time than the open group and there was no conversion to open surgery. There were no differences in the length of ICU stay or hospital stay, or in mortality and complications after surgery. The authors concluded that laparoscopic splenectomy has advantages over open splenectomy and is feasible and safe in hemodynamically stable patients after NOM management failure. Interestingly, in this series, the most common indication for open splenectomy was CT scan findings (73%), whereas the most common indication for laparoscopic splenectomy was NOM failure (90%). Li et al. [44] compared 21 patients who underwent laparoscopic partial splenectomy with 20 patients who underwent laparoscopic splenectomy for traumatic splenic injury. There were no differences in operation time, intraoperative blood loss, or transfusions and there was case of conversion to laparotomy. The two groups were also similar in postoperative complications and hospital length of stay, although the partial splenectomy group had a higher leukocyte count and a lower platelet count 6 months after surgery. This is the only study that compares two different laparoscopic approaches for splenic trauma. Table 2 summarizes the relevant data extracted from these studies.

**Table 2:** Summary of relevant data extracted from published studies

Authors	Year	Type of article	Total cases	Cases treated with laproscopy	Type of performed intervention (laparoscopic	Mean operative time in laparoscopy (min)	Conversi on rate	Mean in hospital LOS (days)	Complications rate	Mortality	Embolization
Nasr et al.	2004	Comparative retrospective study	4	4	Splenectomy: 4; Partial splenectomy: 0; others: 0	175	0%	5.5	25%	0%	0
Huscher et al.	2006	Case series	111	11	Splenectomy: 6; partial splenectomy: 1, others: 4	177	9%	15.2	18.2%	0%	0
Ramson et al.	2009	Comparative retrospective study	11	4	Splenectomy: 4; partial splenectomy: 0; others: 0	140	0%	4.5	0%	0%	11
Carobbi et al.	2010	Case series	12	12	Splenectomy: 10: partial splenectomy: 0; others: 2	115	0%	5.6	8.3%	0%	0
Yahya et al.	2013	Case series	18	18	Splenectomy: 4; partial splenectomy: 0; others: 14	Not reported	5.5%	3.8	0%	0%	0
Huang et al.	2013	Case series	52	11	Splenectomy: 11; partial splenectomy: 0; others: 0	Not reported	Not reported	9.64	9.1%	0%	Not reported
Li et al.	2017	Comparative retrospective study	41	41	Splenectomy: 20; partial splenectomy: 21; others: 0	116	0%	5	19.5%	0%	Not reported
Shamim et al	2018	Comparative retrospective study	25.521	113	Splenectomy: 113; partial splenectomy: 0; others: 0	Not reported	Not reported	9	19.5%	14.2%	Not reported

*LOS* length of stay

Recently, Fransvea et al. [45] published a systematic review of data on 212 laparoscopic splenectomies after trauma. None of the laparoscopic procedures were converted to open surgery, with a mean length of hospital length of stay of 5.85 days and median postoperative morbidity and mortality rates of 14 and 7.5%, respectively. The review concluded, as did the other studies, that laparoscopic splenectomy is a safe and feasible alternative for hemodynamically stable patients after failure of NOM for splenic injury.

## Spleen-preserving laparoscopic surgery for trauma

In the second half of the last century, the discovery of splenic immunologic function led to a gradual shift of the surgical paradigm from splenectomy to splenic conservation. Thus, many surgeons started to perform surgical procedures that preserved the spleen parenchyma, such as splenorraphy and partial splenectomy, and use hemostatic agents. Some authors reported the first laparoscopic procedures for spleen salvage after traumatic injuries.

The laparoscopic use of hemostatic agents and devices have been reported, the most frequent of which is fibrin glue. In 1994, Tricarico et al. [46] reported two cases of splenic trauma treated with laparoscopy with the application of fibrin glue. Surgery led to resolution of the hemorrhage in both patients, although one died 10 days after trauma for concomitant severe brain injury. Two German authors reported another case of splenic injury in a 10-year-old boy managed successfully with the laparoscopic use of fibrin glue [47]. The largest case series of the laparoscopic use of fibrin glue was reported by Olmi et al. [48]. They described the successful application of fibrin glue in six patients, achieving good hemostasis without conversion to open surgery, within a mean operative time of 1 h. In this series, there was no postoperative mortality or morbidity and the mean in-hospital stay was 4.3 days. Other reported hemostatic techniques for splenic salvage include radiofrequency ablation [49], argon beam coagulator [50], and mesh wrapping [34, 51]. Li and colleagues [49] reported a series of four patients, three of whom underwent laparoscopic hemostasis with radiofrequency ablation without surgery, while one was treated with partial splenectomy. One patient required emergency laparotomy and splenectomy for ongoing bleeding, but the others had an uneventful postoperative recovery.

In 1995, Poulin et al. [52] reported a case of partial splenectomy of the superior pole after selective angioembolization of the superior polar artery for splenic injury after blunt trauma. More recently, the case of a 15-year-old boy with upper pole splenic injury was reported [53]. After an initial trial of conservative treatment, the patient underwent laparoscopic exploration of the abdominal cavity because of ongoing bleeding, as indicated by a decrease in the hemoglobin level from 12 to 10 g/dL with an increased heart rate, 4 h after hospitalization. Laparoscopic splenic resection of the upper pole was carried out with an operative time of 150 min, but there were no complications after surgery. The largest number of reported laparoscopic partial splenectomies for traumatic injuries was reported by Li et al. [44].

## Current indications for laparoscopic splenectomy for splenic traumatic injuries

Laparoscopic surgery is gaining importance in the management of abdominal trauma. Many studies have explored this in recent years [4], and shown the advantages of this approach in hemodynamically stable patients. In fact, while trauma patients can benefit from the same advantages of laparoscopy for elective surgery (less abdominal pain, less wound infections, and faster recovery), the risks of both diagnostic and therapeutic laparoscopic surgery for trauma, such as missed injuries or the need for conversion, are limited if minimally invasive surgery is performed by experienced surgeons [54, 55].

According to the available literature as summarized above, laparoscopic splenectomy for traumatic injury seems to be indicated for hemodynamically stable patients when initial NOM management fails because of ongoing bleeding, shown by a continuous drop in hemoglobin and the need for continuous transfusions or infusion of fluids to maintain good vital signs. Other indications can be considered as “special cases”: for example, if patient, after having been adequately informed about the risks and benefits of NOM, refuses conservative treatment in favour of splenectomy or if strategies for successful NOM cannot be adopted, as in the case of a Jehovah’s witness patient who refuses transfusions, or if there are limited resources.

**Fig. 1:**
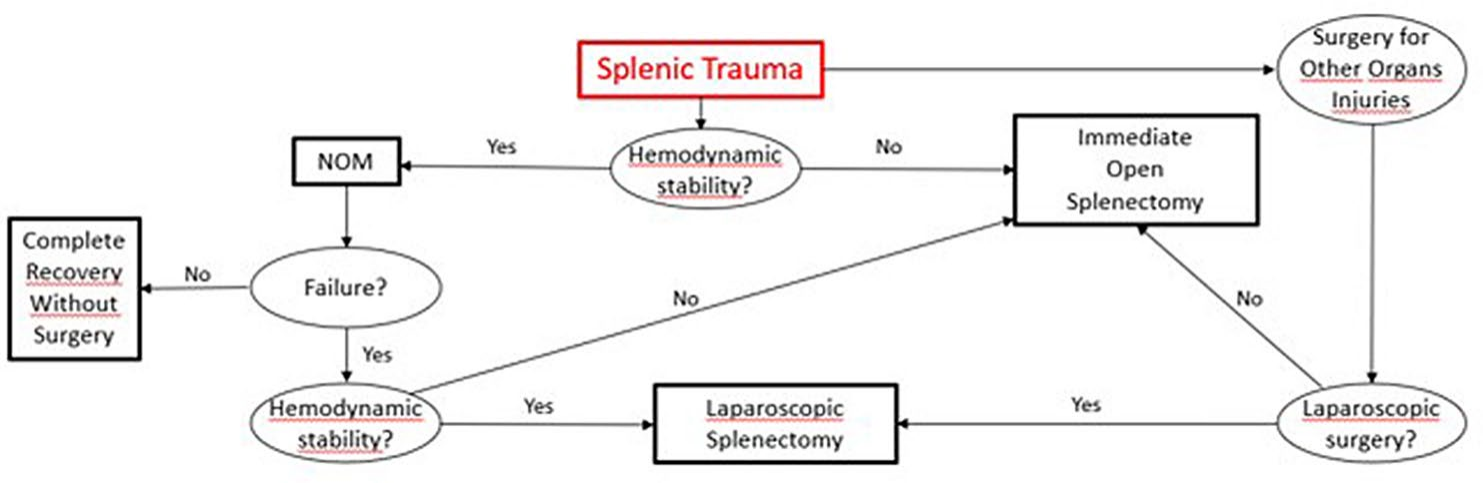
Flow chart showing the current role of laparoscopic splenectomy in the treatment of splenic traumatic injuries. Currently, laparoscopic splenectomy is indicated only for hemodynamically stable patients when non-operative management has failed. If laparoscopic surgery is being performed for other abdominal organ injury, laparoscopic splenectomy may be considered when an associated splenic injury is found

The current WSES guidelines [11] suggest that immediate splenectomy should be performed for splenic injury with hemodynamic instability or if surgery for injuries of other abdominal organs is required. The more frequent application of laparoscopic surgery for many abdominal traumatic injuries, could pave the way for laparoscopic splenectomy even for patients requiring laparoscopic surgery for other traumatic injuries. Figure 1 proposes a treatment strategy for splenic trauma, considering laparoscopic splenectomy.

## Future perspectives of laparoscopic splenectomy for splenic trauma

**Fig. 2:**
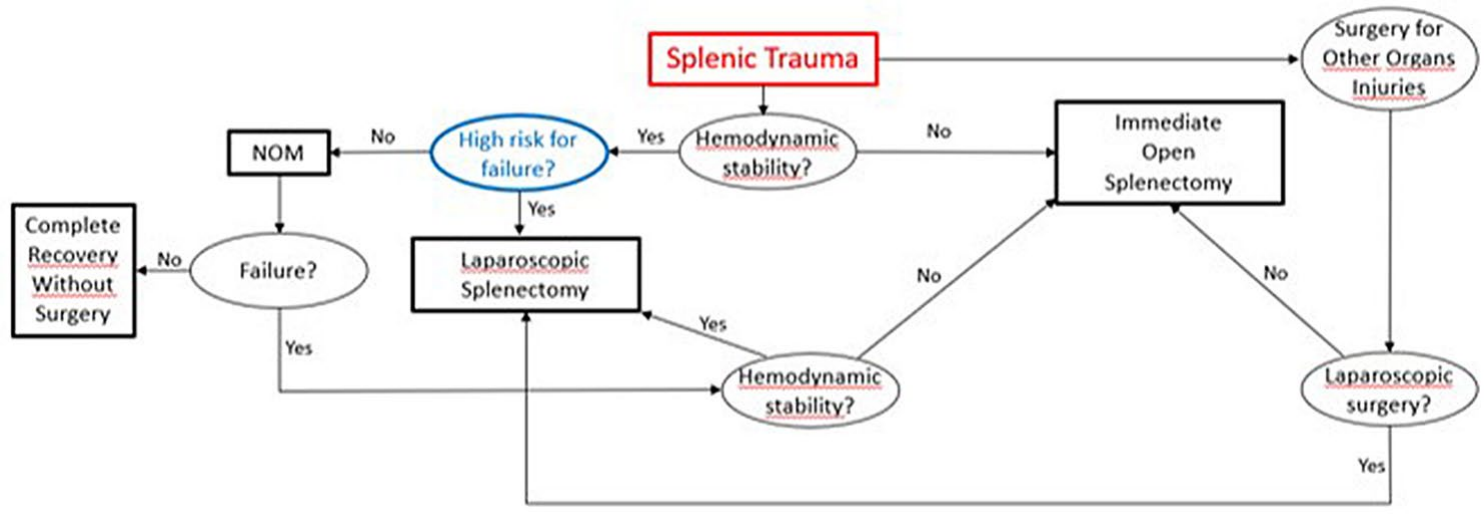
Our hypothetical strategy for the treatment of splenic traumatic injury, if a reliable prediction of non-operative management failure risk can be made: Risk stratification could lead to a diversification of treatment for high-risk splenic injuries from low-risk injuries in hemodynamically stable patients

Nonoperative management is the treatment of choice for hemodynamically stable patients with traumatic splenic injury; however, high-grade injuries are associated with higher failure rates, even if the adjunct of angioembolization can reduce this rate. Although many studies have tried to identify which indicators are reliable predictors, there are no clear outcome predictors and no prognostic assessment tools to help surgeons select patients for NOM after splenic injury. In fact, patients presenting with a high risk of failure of NOM, and, therefore, at risk of morbidity and mortality, may be candidates for immediate surgical treatment. Given this, the development of tools for the prognostic stratification of these patients could lead to novel strategies to treat splenic traumatic injuries. In this context, we anticipate that in the future, along with a better definition of failure risk of NOM, the indications for minimally invasive splenectomy could be expanded to include patients presenting with a high risk of failure of NOM, leading to better results in the overall treatment of traumatic injuries of the spleen. Figure 2 shows our prediction of how the treatment strategy for splenic trauma will change if the risk of NOM failure can be better assessed.

Another important issue to consider is the infection risk to which splenectomized patients are exposed. The reported incidence of OPSI varies in different series [56]. However, most series on post-splenectomy infection are dated, so the true incidence of infection and mortality rates are unclear. A recent analysis reported a low incidence of OPSI after splenectomy, even with a mortality rate of about 50% [57]. Moreover, the adherence to immunization protocols is still limited [58–60] and the need for revaccination after the first dose is still matter of discussion [61]. The implementation of vaccination protocols and an active education program for patients treated with splenectomy may lower the risk of infective complications of patients undergoing splenectomy for trauma. Another factor influencing the risk of severe infections after splenectomy is disease necessitating surgery, with trauma patients appearing to be at lower risk than hematological and oncological patients [57]. Thus, the current incidence of post-splenectomy infection and its mortality rate in trauma patients could be reduced with complete adherence to vaccination protocols.

This prompts the question: is there a greater risk of NOM failure or of developing OPSI? A correct assessment of NOM failure risk is a key element in answering this open question and in deciding whether to perform a splenectomy or initiating NOM.

## The future of laparoscopic spleen-preserving surgery?

Since the introduction of NOM, the role of spleen-sparing surgical treatment of splenic injuries has been reappraised, and is almost forgotten in adult surgery. Conversely, in pediatric trauma surgery, splenorraphy and partial splenectomy are the preferred treatment for hemodynamically unstable patients who require immediate surgery to control bleeding [11, 62], because of the higher susceptibility of children to post-splenectomy sepsis. Considering this, it is difficult to imagine that laparoscopic conservative surgery of the spleen will find its place in the future, except in highly selected cases, since it is reserved for hemodynamically unstable pediatric patients for whom laparoscopic surgery is not considered safe.

## Conclusion

Laparoscopic surgery for the treatment of hemodynamically stable splenic injury seems feasible according to the available literature. Some retrospective studies comparing open and laparoscopic splenectomies showed that the laparoscopic approach has better short-term outcomes. NOM is widely accepted for low-grade splenic injuries and despite it being recommended for hemodynamically stable high-grade splenic injury, it has a high failure rate in this subgroup. Against this background, the pros and cons of NOM should be considered carefully for each patient. Laparoscopic surgery for hemodynamically stable splenic trauma is now a good alternative to open surgery when performed by an experienced surgeon, if NOM fails, and should be encouraged. Future knowledge will allow surgeons to predict NOM failure more precisely and laparoscopic surgery could become the treatment of choice for those patients at high risk of its failure.

This review summarizes the current knowledge of the laparoscopic treatment of splenic injuries and should not be taken as a guideline. We also tried to envisage what developments could change physicians’ attitudes to the treatment of splenic trauma in the future, in the hope of stimulating scientific debate.
